# The Role of Data in Public Health and Health Innovation: Perspectives on Social Determinants of Health, Community-Based Data Approaches, and AI

**DOI:** 10.2196/78794

**Published:** 2025-10-07

**Authors:** Kaakpema Yelpaala, Michael Christopher Gibbons, Ines Maria Vigil, Jennifer Leaño, Terika McCall, Ijeoma Opara, Anne Zink, Marcella Nunez-Smith, Bhramar Mukherjee, Megan Ranney

**Affiliations:** 1 Department of Health Policy and Management, Yale School of Public Health, 60 College Street, New Haven, CT, 06510, United States, 1 2037852867; 2 The Greystone Group, Greenbelt, MD, United States; 3 Healthcare Management and Administration, University of Michigan School of Public Health, Ann Arbor, MI, United States; 4 Yale School of Public Health, New Haven, CT, United States; 5 Department of Biostatistics, Yale School of Public Health, New Haven, CT, United States; 6 Department of Social and Behavioral Sciences, Yale School of Public Health, New Haven, CT, United States; 7 Section of General Internal Medicine, Yale School of Medicine, New Haven, CT, United States

**Keywords:** social determinants of health, SDOH, community-based data approaches, artificial intelligence, AI, data equity, health policy, Health Insurance Portability and Accountability Act, HIPAA

## Abstract

Public health is undergoing profound transformation driven by data from the global health sector and related fields. To address systemic health disparities, scholars and health practitioners are increasingly applying a data equity lens, an approach that has become even more urgent as the United States faces the erosion of public health data infrastructure. This paper summarizes insights from an April 2024 convening by the Yale School of Public Health—The Role of Data in Public Health Equity and Innovation—with intersectoral stakeholders from academia, government (local, state, and federal), health care, and private industry. The convening included keynote presentations and roundtables regarding the depiction of social determinants of health in data; effects of artificial intelligence (AI) on health data equity; and community-based models for data, providing a framework for cross-cutting discussions. Through a narrative synthesis, themes were identified and synthesized from systematically gathered information from presentations and roundtables. This process led to a set of actionable, cross-cutting recommendations to guide inclusive and impactful data practices for policymakers, public health professionals, and health innovators across diverse contexts: (1) enable big data and interoperability connecting social determinants of health and health outcomes; (2) include diverse, nontechnical voices in AI and health discussions; (3) fund research on data equity and AI in health sciences; (4) modernize the Health Insurance Portability and Accountability Act (HIPAA) with new guidelines for AI and big data; and (5) research and conceptual frameworks are needed to elucidate interconnections between data equity and health equity.

## Introduction

The public health landscape is undergoing profound transformation driven by vast amounts of data generated through global digital transformation of the health sector and related fields [1]. Data science enhancements, novel biostatistical methods, genomics, and artificial intelligence (AI) methods coupled with multimodal datasets (ie, imaging and wearables) accelerate this evolution [2]. This offers unprecedented opportunities to advance health and human welfare through public health research and practice. These advancements also bring significant challenges, particularly the risk of exacerbating systemic health disparities and producing adverse unintended consequences. To address these concerns, scholars and practitioners are increasingly applying the lens of data equity. The World Economic Forum defines data equity as “the shared responsibility for fair data practices that respect and promote human rights, opportunity and dignity” [3]. This paradigm is not only critical to ensuring that the innovations in public health are equitable and ethical but also timely in light of the ongoing dismantling of public health data infrastructure and persistent challenges related to the fragmentation of health information systems in the United States.

This viewpoint paper offers a narrative synthesis and policy perspectives based on structured expert dialogue rather than original empirical research. We summarize insights from a conference hosted by the Yale School of Public Health in April 2024 exploring data equity, public health, and health innovation. These themes remain highly relevant amid shifting national priorities in the United States and increasing pressure on public health data systems. We present actionable, interdisciplinary recommendations to guide researchers and practitioners in embedding equity-based principles into data collection, analysis, and insight generation. Our goal is to empower health policymakers, public health professionals, data scientists, and health innovators to adopt inclusive and impactful data practices across diverse contexts.  

## Conference Structure and Approach

Our team comprised the multidisciplinary steering committee of faculty and researchers with public health, digital health, health equity, and clinical backgrounds who developed the structure and central theme of the conference, The Role of Data in Public Health Equity and Innovation. The conference focused on 3 interrelated thematic areas for keynote presentations and roundtable discussions: the depiction of social determinants of health (SDOH) in data, the effect of AI on health data equity, and community-based models for data.

These thematic areas provided a comprehensive framework to facilitate discussions on cross-cutting issues related to data equity in public health. This effort culminated in a 2-day conference on April 8 and 9, 2024, which brought together a select group of invited guests representing public health leaders and community advocates from academia, government (local, state, and federal), health care, and private industry.

The conference agenda was divided into 2 parts: day 1 was focused on keynote presentations, moderated panel discussions on each thematic area, and a fireside chat; day 2 was focused on half-day roundtable discussions dedicated to each thematic area.

Attendees could participate in up to 2 roundtable discussions. Each session was supported by a facilitator and 2 graduate student notetakers. Facilitators opened with a framing of the thematic area, followed by moderated discussions. Sessions concluded with a summary of key points, which participants refined and collaboratively prioritized. Notetakers captured these insights in real time.

With their diverse expertise in public health research, practice, policy, community engagement, and health equity, our team—comprising faculty and researchers with public health, digital health, health equity, and clinical backgrounds—systematically organized the notes by theme. They cross-checked summaries, integrated relevant peer-reviewed literature, and iteratively refined key themes to ensure accuracy and reduce bias. This synthesis process produced actionable, cross-cutting findings and recommendations that reflect the diversity of perspectives represented at the convening. While the convening included discussion of community-based data models, community members were not directly involved in the roundtable synthesis process. Recognizing this limitation, future convenings should more intentionally include community representatives as participants and cocreators in the synthesis and recommendation development process.

## Key Findings From the Roundtable Discussions

This section highlights the central topics discussed during each roundtable session. Each thematic area is explored in detail, followed by key insights and takeaways from the convening. The insights aim to provide actionable guidance and a deeper understanding of the issues related to data equity in public health.

### The Depiction of SDOH in Data

SDOH—nonmedical factors that influence health outcomes and encompass conditions where people are born, grow, live, work, and age, in addition to race, ethnicity, and language—are typically categorized into 5 domains: economic stability, education access and quality, health care access and quality, neighborhood and built environment, and social and community context [4,5]. The profound impact of SDOH accounts for up to 80% of health-related outcomes, compared to the roughly 20% attributed to clinical care [6].

To better understand connections between SDOH and health outcomes, it is essential to integrate longitudinal data sources beyond clinical data from electronic health records (EHRs) [7]. Growing interest in capturing SDOH data reflects its importance in promoting health equity. Examples include the Centers for Medicare and Medicaid Services Center for Medicare and Medicaid Innovation’s Health-Related Social Needs Screening Tool and the American Academy of Family Physicians social needs screening tool for family medicine practices [8,9]. Innovations such as large language models (LLMs) to identify SDOH in EHR narrative text are being developed [10]. Z-codes have been added to the *International Classification of Diseases, 10th Revision* allowing for social service reimbursement [11]. Despite advancements, most SDOH data reside outside of the health care setting, making collection, standardization, and analysis a challenging but critical opportunity.

#### SDOH Roundtable Discussions

Participants explored the following SDOH-related topics: (1) the role of big data, (2) the need to break down silos between data sources, and (3) methods to improve interoperability across data systems. In this context, “big data” refers to the volume of structured and unstructured data generated by diverse digital sources [12]. Recognizing the limitations of structured SDOH data collection within health care workflows, participants highlighted the potential of alternative data sources such as social media, mobile applications, wearables, and digital imaging to deepen insights into social influences on health outcomes. Big data, coupled with machine learning (ML) and predictive analytics, offers the potential to model the complex and bidirectional relationship between social factors and health outcomes and provide insight into implementing and delivering interventions with greater precision [13,14]. Such insights could influence policies and interventions that address the root causes of health disparities.

Another significant topic was interoperability—seamless data integration from various sources—to harness SDOH data effectively [15]. Ethical considerations, including ensuring informed consent from individuals for data use, were also emphasized, along with the importance of clearly defining the purpose and intent of SDOH data collection.

#### Promising Initiatives

Participants shared practical approaches to integrating SDOH data. Notably, Colorado Office of eHealth Innovation representatives discussed their efforts in launching a first-of-its-kind statewide initiative improving health outcomes by integrating SDOH and health care information, the Colorado Social Health Information Exchange (CoSHIE) [16]. This secure, centralized platform facilitates data sharing among stakeholders (ie, health care providers and social service organizations), enabling coordinated, holistic social and clinical care. Promoting advanced data interoperability and robust privacy protocols, CoSHIE empowers service health care providers to deliver targeted, impactful interventions, enhance patient experiences, and reduce health care costs, in addition to facilitating cross-sector communication and referrals, addressing social and health issues collaboratively and effectively.

#### Key Insights

##### Broadening the Data Scope

Understanding the root causes of health challenges requires integrating SDOH data from diverse sources beyond clinical data and traditional surveys. Big data expands the breadth of information available, offering new opportunities to understand differential health outcomes across populations. Further research is needed to establish best practices regarding privacy in the collection, compilation, and use of SDOH and big data for both researchers and practitioners.

##### Interoperability Challenges

Significant barriers remain in integrating SDOH data, including non–EHR-based datasets, into enhancements in health care, social care, and broader population health. While state-level initiatives such as CoSHIE are promising, broader interoperability challenges exist at the local, state, and federal levels. Approaches such as the Trusted Exchange Framework and Common Agreement, a nationwide US framework for secure health information exchange, are facilitating interoperability among organizations that opt in to the program. Data use consent, privacy, and data-sharing policies require careful consideration, particularly when data cross state or federal boundaries.

##### AI and ML in the Context of SDOH and Big Data

AI and ML offer transformative potential to rapidly analyze connections between social factors and health outcomes, including the big data themes discussed above. However, more research is needed to identify effective tools, methodologies, and applications for these technologies in the SDOH context.

### The Effect of AI on Health Data Equity

Data equity involves addressing biases in data systems, ensuring diverse and culturally sensitive methodologies, and empowering communities to have a voice in how their data are used. It helps identify and address health disparities, design interventions responsive to the needs of different populations, and build trust with communities. By prioritizing equitable data practices, stakeholders can create more effective, inclusive health policies and programs that promote fairness and better health for all.

The rapid evolution of AI has immense potential to drive health care and biotechnology transformation. Data equity concerns regarding its real-world impacts include the risks of amplifying health disparities and unequal distributions of benefits across populations. For example, while researchers are in the early stages of documenting LLM applications in health care and public health, these models can also perpetuate biases, potentially exacerbating health inequities [17,18]. LLMs currently perform better in English than other languages, posing challenges to equitable global adoption while risking reinforcement of systemic biases in health care, justice, and safety systems [19,20]. Participants emphasized the urgency of addressing these biases. Strategies included creating indexes to assess bias in model performance and integrating community perspectives into AI development to foster trust and empowerment [20-22]. One participant who coauthored the A.C.C.E.S.S. AI model (affirm your aims [why], consider your communities [who], cultivate your conversation [when], embrace your essentials [how], specify your scope [what], and scrutinize your space [where]) highlighted it as a toolkit for advancing AI health equity [23]. The A.C.C.E.S.S. AI model ensures community engagement in AI and accurately represents diverse populations through data. Similar guidelines for trustworthy use of AI and model transparency include the Transparent Reporting of a multivariate prediction model for Individual Prognosis or Diagnosis Statement and the Coalition’s for Health AI’s Health AI Assurance Standards Guide [24,25]. Participants stressed that AI models are only as inclusive as the data they use.

#### Inclusion and Unlearning

Inclusion and representation were central to discussions, alongside the concept of “machine unlearning.” This is a theoretical approach in generative AI that explores how models might “forget” or adjust elements of historical knowledge, including embedded biases such as those linked to race or gender [26,27]. This idea draws parallels to the “right to be forgotten” provision in the European Union’s General Data Protection Regulation, which gives individuals the right to request the deletion of personal data [28]. While machine unlearning remains largely experimental and, to the participants’ knowledge, has not been applied in public health contexts, it reflects a growing interest in embedding data equity at the systems level. As AI becomes more integrated into public health decision-making, the ability of models to unlearn, if developed responsibly, could have meaningful implications for addressing structural bias in digital health systems.

Other techniques discussed included fine-tuning AI models. The process involves training models with an established understanding of language or tasks on a specific dataset to enhance performance on that specific task or domain [29]. While promising, these approaches come with technical challenges, cost implications, and the risk of propagating new inaccuracies [30-32]. Participants noted that bias is inherently linked to human behavior and cognition, and since AI tools are created by humans and trained on human-generated data, there is a risk of replacing one set of biases with another. Crucially, not all biases are equally problematic in the context of health care; the most concerning are those with the potential to cause real-world harm to individuals or communities.

#### Open-Source Versus Proprietary Models

Participants debated the merits of open source versus proprietary AI models. Advocates highlighted the transparency, user control, and collaborative potential of open-source models such as Meta’s LLaMA and Meditron, a clinical language model suite built on LLaMA and adapted for low-resource medical settings. [33,34]. They emphasized the promise of these models for innovation in low- and middle-income countries. Critics, however, noted that such models rely on infrastructure from major technology companies and face challenges related to security, quality control, and feature development. Meanwhile, proponents of proprietary AI models emphasized their potential for rapid innovation, although with trade-offs in accessibility and inclusivity.

#### Building Trust and Multidisciplinary Governance

In light of past missteps by health care and technology actors, participants underscored the importance of transparency and trust-building between AI developers and the communities they serve. Enhancing AI literacy through community-engaged initiatives and integrating AI into educational curriculum was identified as a crucial step to ensure that AI is developed *with* communities, not merely *for* them. Participatory approaches to AI governance were recommended, involving multidisciplinary experts from fields including ethics, behavioral and social sciences, and anthropology. This approach would democratize access to AI knowledge and ensure diverse perspectives and ethical oversight.

#### Connecting Data Equity to Health Equity

The need for robust frameworks connecting data equity principles [35,36] to health equity was a recurring theme. Participants emphasized meaningful equity in AI requires more than technical fixes; it demands collaborative, intersectoral efforts bridging health equity expertise with AI innovation. Traditional definitions of equity in addressing the unique ethical and policy challenges posed by AI underscore the need for new conceptual frameworks to guide this work [37].

#### Key Insights

##### Fine-Tuning Generative AI Models

Fine-tuning AI models using diverse health datasets is a promising avenue to mitigate bias, reduce hallucinations, and enhance accuracy and trustworthiness. Model transparency frameworks also play an important role in the development of equitable AI systems. However, further research is needed to optimize these methods while balancing cost efficiency.

##### Challenges in Data Equity and Generative AI

Applying data equity principles to generative AI presents challenges, including safeguarding data privacy and addressing model hallucinations. If a model performs poorly for underrepresented groups, its predictions for these groups may degrade faster than for others as conditions change. Model drift can amplify bias when deployed environments shift disproportionately across populations, and conversely, bias in training data can trigger drift by degrading model performance for certain demographic groups [38,39]. In other words, if a model is trained on biased data, it begins making inaccurate predictions. As real-world conditions and data change, those gaps often widen, especially for groups that were underrepresented. Clear frameworks are essential and needed to connect data equity with health equity and to provide agile guidance for researchers and practitioners working at the intersection of these fields.

##### Data Sovereignty and Community Engagement

Issues of data sovereignty, ownership, and extractive data practices threaten data equity in AI-driven health solutions. Existing ethical standards are inadequate to address these challenges. Ethical standards must evolve to build trust, reliability, and effectiveness. Engaging communities through participatory research is important to codevelop strategies that balance equity, fairness, and accuracy in the use of health data. Furthermore, equity can enhance the accuracy of models, enhancing health innovation for all beneficiaries.

### Community-Based Models for Data

Community-based data models are participatory frameworks designed to generate localized, actionable insights that improve health outcomes and address public health challenges. Actively involving community members to define priorities, collect data, and shape initiatives ensures that data practices reflect the needs, values, and lived experiences of diverse populations. They are critical for deploying equitable and relevant resources that improve health outcomes. They influence patient behaviors and outcomes while identifying blind spots in research and practice [40,41]. Persistent barriers limit effective data sharing with the very communities from which it is collected, thereby reducing the impact of these insights and diminishing a community’s agency over its own health [42]. Participants identified 3 central themes to actualize community-based data model potential.

#### Defining Community

“Community” cannot be defined using a one-size-fits-all approach. Communities can be shaped by geographic region, ethnicity, or shared experiences and intersecting identities, complicating efforts to capture multiple layers of diversity using only quantitative data [43]. Cocreating definitions and methods with communities ensures that data collection reflects community needs and values while fostering collective data stewardship.

#### Promoting Equity and Collaboration

Collaboration among stakeholders—community members, researchers, and policymakers—is essential to ensure reciprocity and equity in data practices. Historical power imbalances have led to mistrust in institutions, causing hesitation in sharing information. Policymakers, public health practitioners, and technologists must address these concerns by designing community engagement approaches prioritizing transparency. Engaging communities in discussions about data ownership, consent, and intended use fosters trust and ensures that initiatives align with their expectations.

#### Enhancing Representation

Traditional quantitative methods often fail to capture the full spectrum of community experiences, while standardized data collection can oversimplify complex realities. Mixed methods approaches combining quantitative and qualitative data provide a more comprehensive understanding of community needs. This approach empowers communities to define their own identities and ensures that data-driven actions genuinely reflect their lived experiences. Qualitative approaches continue to have importance as we expand our quantitative data approaches through AI.

A multifaceted approach to community-based data collection and participatory approaches is key to promoting data equity in public health. Acknowledging that communities may be defined in numerous ways, this approach grants community members the autonomy to define their identities, ensuring data that genuinely represent their experiences. This process faces challenges, including historical power imbalances that led to mistrust in institutions, causing some communities to hesitate to share information. Policymakers, public health practitioners, and technologists must consider multifaceted approaches when designing community-engaged methods for data collection and use. Conference participants suggested that it is important to incorporate quantitative and qualitative methodologies in research at the intersection of data equity and population health. Ethical concerns around data ownership, privacy, and consent must be addressed early in program design to protect participants’ integrity and agency.

### Building Trust and Empowering Communities

Trust and transparency were recurring themes in roundtable discussions. Clear communication about data collection processes, use, and sharing is crucial. Participants stressed the importance of equitable data practices that give diverse communities a voice in shaping data initiatives. Mixed methods approaches that combine quantitative and qualitative data portray fuller pictures of community needs, empowering communities to take ownership of their data. To ensure engagement and trust, communities should also benefit directly from the data they share, whether through financial resources, enhanced services, or actionable insights that improve their well-being. DataHaven, a nonprofit organization that provides reports and data to inform practice and policies, is one example of how organizations and communities can partner using data-driven strategies to enhance community well-being [44].

As AI becomes more integrated into public health, ensuring that models are transparent, interpretable, and safeguarded against or free from bias is critical. Decentralized data models, where communities retain control over their data, further reinforce data sovereignty and empower local stakeholders. These models foster trust and strengthen the ability of communities to influence health interventions and public health research and practice.

### Key Insights

#### Acknowledging Diversity in Communities

Community-based data models must account for diverse and intersecting identities within communities. There is no universal definition of “community,” and understanding its complexities requires cocreating definitions and methodologies with community members. This ensures that data reflect the unique needs and values of diverse populations. Additional research is needed to establish best practices for community-based data models in developing public health interventions and AI apps.

#### Addressing Mistrust and Historical Imbalances

Historical power imbalances and institutional mistrust pose challenges to community participation in data-sharing initiatives. Transparent communication about data collection, use, and benefits, coupled with measures to address privacy, consent, and data ownership concerns, is essential for fostering trust. Equitable and inclusive data practices rooted in community engagement can center principles of data equity, particularly in the context of SDOH, big data, and applications of AI-driven solutions benefiting community health.

#### Leveraging Mixed Methods and Decentralized Models

Even with advances in big data, generative AI, and novel biostatistical approaches, combining quantitative and qualitative data provides a richer understanding of community needs, ensuring more inclusive representation. Decentralized data models, where communities maintain control over their data, promote sovereignty and empowerment, reinforcing trust and collaboration at the local level. These models offer a promising path to sustainable, data-driven public health initiatives at a local level.

## Implications for Policymakers and Practitioners

Drawing from the insights and discussions of the conference, we outline 5 actionable, cross-cutting recommendations for policymakers and practitioners in health-related fields.

The first recommendation is to enable big data and interoperability, as they are central in connecting SDOH and health outcomes. The growing scope of available data to understand the influence of SDOH on health outcomes provides opportunities to address health disparities. However, data standardization, informed consent, data-sharing protocols, and interoperability remain critical challenges. Connecting health information exchanges from EHRs with other multifaceted datasets while ensuring data quality is essential for realizing this potential. Initiatives such as CoSHIE exemplify how states can advance interoperability and SDOH data integration.

The second recommendation is to include diverse, nontechnical voices in AI and health discussions. As AI governance frameworks and ethical guidelines evolve, integrating perspectives beyond technical and medical expertise is crucial. In the absence of clear federal regulation in the United States and with a patchwork of varying state AI bills, some groups are proactively setting clear guidelines on the ethical use of AI in their context. We observe that many of these forums in the health sector include engineering, data science, and medical perspectives but often exclude other voices. Varied sociotechnical perspectives and nontechnical voices representing social sciences, community leaders, and advocates should be engaged to develop well-rounded perspectives on the use of AI in the health context. It should be the responsibility of the group convening such a committee to ensure the appropriate level of AI literacy for diverse stakeholders to engage in a substantive manner.

The third recommendation is to foster collaboration between the academic institutions, the private sector, and the local systems to support data equity in the health sector. With the unwinding of public health data infrastructure in the United States, there is an opportunity to increase collaborative efforts between academic institutions, the private sector, and other local actors to protect, expand, and steward data for public health and health innovation. Privacy and building and maintaining trust with those who share data will be central to this endeavor. Local and state governments can promote responsible AI development that prioritizes local control and agency over data. From the perspective of academic institutions and the private sector, it will be important to identify an approach that facilitates academic integrity and mitigates conflicts of interest while being relevant to the scale at which many corporations operate.

The fourth recommendation is to modernize the Health Insurance Portability and Accountability Act (HIPAA) and develop new guidelines for AI and big data. To address the unique challenges posed by the integration of AI in health care, HIPAA and related regulatory frameworks must be modernized. As well, complementary regulations need to be created to address the risks and opportunities posed by AI and big data in the health sector. Current laws with a focus on patient privacy and data security leave gaps in oversight for AI-driven applications. Modernization efforts should include provisions for algorithmic transparency, bias mitigation, and data equity, ensuring these technologies are used ethically and equitably. Clear and robust AI-tailored guidelines are essential for balancing innovation with ethical and equitable health care practices, ultimately enhancing patient outcomes and system reliability. Collaboration among legal experts, health policymakers, health care providers, public health practitioners, and other stakeholders’ community representatives is essential to create robust guidelines.

The fifth recommendation is that research and conceptual frameworks are needed to elucidate the interconnections between data equity, AI, and improving health outcomes. There is a need for new frameworks that explicitly connect data equity, AI, and improving health outcomes. Traditional approaches using data to improve health outcomes may overlook the critical role of data equity in identifying and addressing disparities, while data systems may fail to account for structural inequities that shape health outcomes. Integrative frameworks can illuminate how biases in data collection, analysis, and application perpetuate inequities, enabling stakeholders to design interventions that address root causes rather than symptoms. These frameworks can guide the ethical use of emerging technologies, such as AI and digital health tools, to be equitable and inclusive. They also facilitate impactful collaboration between policymakers, researchers, and communities.

## Conclusions

The Yale School of Public Health conference on data equity and health innovation highlighted the critical role of equitable data practices in shaping the future of public health, particularly in the current context of public health data in the United States. Achieving health data equity requires a holistic approach that extends beyond conventional practices to embed equitable principles across all stages of development and policymaking, driven by interdisciplinary collaboration and meaningful community engagement. The transformative potential of leveraging big data, community-driven models, and AI to improve population health outcomes emphasizes the need to break down silos between datasets, enhance interoperability, and foster ethical AI applications that prioritize inclusivity and mitigate biases.

Policymakers and practitioners have a unique opportunity to embed data equity principles into every stage of data and AI development at this pivotal juncture in public health. Data equity should not be treated as a peripheral consideration but as a guiding framework. Achieving this requires meaningful community engagement, transparency to foster trust, and integration of diverse perspectives into both policy and technological design. While fairness metrics are often used to operationalize equity within AI systems, equity encompasses a broader, more nuanced concept—one that ensures inclusive data collection and system design that distributes benefits fairly and mitigates harm, particularly with respect to health outcomes. Operationalizing equity requires measurable accountability mechanisms such as model auditing tools, participatory governance structures, and dashboards that track differential impacts on populations over time.

Importantly, these recommendations must also be understood in the context of shifting public health priorities, constrained infrastructure, and tightening fiscal environments, particularly as Medicaid funding declines and many states face significant budgetary pressures. These structural challenges will likely hinder the implementation of equity-centered data strategies, especially in underresourced jurisdictions. Recognizing these headwinds reinforces the urgency of advancing coordinated, scalable, and inclusive approaches to data and innovation through novel partnerships that can adapt to resource variability while maintaining a commitment to equitable health outcomes.

The conference provided a foundation for future research and action, reinforcing the importance of interdisciplinary collaboration to ensure that advancements in data and AI translate into equitable health outcomes. By broadening the lens through which equity is understood and applied, stakeholders can drive meaningful progress at the intersection of technology, policy, and public health, ensuring that innovation results in tangible benefits for all communities.

## Abbreviations

A.C.C.E.S.S.: affirm your aims (why), consider your communities (who), cultivate your conversation (when), embrace your essentials (how), specify your scope (what), and scrutinize your space (where)
AI: artificial intelligence
CoSHIE: Colorado Social Health Information Exchange
EHR: electronic health record
HIPAA: Health Insurance Portability and Accountability Act
LLM: large language model
ML: machine learning
SDOH: social determinants of health
